# Fire Ant Venom Alkaloids: Possible Control Measure for Soilborne and Foliar Plant Pathogens

**DOI:** 10.3390/pathogens10060659

**Published:** 2021-05-27

**Authors:** Sujan Dawadi, Fulya Baysal-Gurel, Karla M. Addesso, Prabha Liyanapathiranage, Terri Simmons

**Affiliations:** 1 Entomology Department, Purdue University, 901 W State Street, West Lafayette, IN 47907, USA; sdawadi@purdue.edu; 2 Otis L. Floyd Nursery Research Center, Department of Agricultural and Environmental Sciences, Tennessee State University, McMinnville, TN 37110, USA; kaddesso@Tnstate.edu (K.M.A.); simmons37352@yahoo.com (T.S.); 3 Department of Entomology and Plant Pathology, Auburn University, 301 Funchess Hall, Auburn, AL 36849, USA; pdl0007@auburn.edu

**Keywords:** soilborne pathogens, foliar pathogens, red imported fire ant, venom alkaloids, alarm pheromone, inhibitory zone, natural products

## Abstract

The purpose of this study was to evaluate fire ant venom alkaloids and an alarm pheromone analog against several plant pathogens, including *Botrytis cinerea*, *Fusarium oxysporum*, *Phytophthora nicotianae, P. cryptogea*, *Pseudomonas syringae*, *Phytopythium citrinum*, *Rhizoctonia solani*, *Sclerotonia rolfsii*, *Xanthomonas axonopodis*, and *X. campestris*. All pathogens were tested against red imported fire ant venom alkaloid extract and alarm pheromone compound for growth inhibition in in vitro assay. The venom alkaloid extract inhibited fungal and oomycete pathogens. Neither of the treatments were effective against bacterial pathogens. Three soilborne pathogens, *P. nicotianae*, *R. solani*, *F. oxysporum*, and one foliar pathogen, *B. cinerea* were selected for further in-vivo assays on impatiens (*Impatiens walleriana* ‘Super Elfin XP violet’). Total plant and root weight were higher in venom alkaloid treated plants compared to an inoculated control. The venom alkaloid treatment reduced damping-off, root rot severity, and pathogen recovery in soilborne pathogen inoculated plants. Similarly, venom alkaloid reduced Botrytis blight. However, higher venom rates caused foliar phytotoxicity on plants. Therefore, additional work is needed to evaluate rates of venom alkaloids or formulations to eliminate negative impacts on plants. Overall, these results suggest that red imported fire ant venom alkaloids may provide a basis for new products to control soilborne and foliar plant pathogens.

## 1. Introduction

One of the major limiting factors in plant production systems is a disease. Soilborne pathogens such as *Fusarium oxysporum*, *Rhizoctonia solani*, and *Sclerotinia rolfsii* are devastating pathogens of cereals [1], vegetables [2], and ornamental production systems [3]. *Rhizoctonia solani*, for example, can cause pre- and post-emergence damping-off, killing seedlings prior to or shortly after emergence. Plant mortality may also be caused by subsequent stem rot and foliar blights [4]. Oomycete plant pathogens, i.e., *Phytophthora* spp., are transmitted through infected soil, water, plant tissue parts, and debris [5]. *Phytophthora nicotianae*, one of the most devastating oomycetes, is found in soils across the southeastern US [6,7] and is associated with 255 genera of 90 plant families [8]. The fungal pathogen, *Botrytis cinerea*, called gray mold, is also a serious problem worldwide and can infect more than 200 crop species [9,10]. This pathogen is more problematic when horticultural crops are harvested and transported. However, substantial losses caused by *B. cinerea* have been reported in field and greenhouse conditions [9,10]. Bacterial pathogens are another common problem in plant production systems and can be transmitted through rain splash or carried by humans, birds, and insects [11]. Bacterial pathogens commonly spread through the crop production system via contaminated cutting and pruning tools [12].

Chemical fungicides [13] and bactericides [12] are the primary methods used to control plant pathogens in cropping systems globally. Additional techniques available to manage losses to plant pathogens include proper sanitation [14,15], use of resistant varieties/cultivars [14], incorporation of cover crops [3,15], soil solarization [7], biofumigation [16,17], organic amendments [3], soil fertility enhancement [18], soilless culture [19], and biological products [20,21,22,23,24]. The use of a single approach to plant pathogen management is rarely effective. A system-based approach incorporating various management tools is often necessary for the effective suppression of plant pathogens [25].

In the search for new tools for plant disease management, one area of research is the use of natural products and metabolites [26]. Natural products are those that can be extracted from plants or animals [27] and used to control pests [28]. Imported fire ants (*Solenopsis invicta* Buren, *Solenopsis richteri* Buren, and their hybrid; Hymenoptera: Formicidae) invaded the United States almost a century ago and are found to be problematic to human health and agricultural production systems. These ants produce a complex venom, consisting of alkaloids and peptides [29,30,31,32]. Extracts of the venom alkaloids have shown promising results as an antimicrobial [26,33,34,35]. The venom consists of two groups of structurally similar alkaloids: piperidines and piperideines [26,30,31]. One team [26] evaluated the alkaloid classes separately as treatments to manage *Pythium ultimum*. The authors found no difference between the performance of the two alkaloid types. The inhibitory effects of fire ant venoms observed for plant and human pathogens lasted for at least 24–48 h, which was the evaluation length of most previous trials [26,34,35]. Since pathogens have differential rates of growth, the effectiveness of a treatment may change over time. Additionally, previous research on plant pathogens was conducted under lab conditions only, not on crop plants.

To fill up the gaps, the in vivo and in vitro assays were conducted to determine if the fire ant venom alkaloid extract and/or alarm pheromone can control key plant pathogens found in nursery crops. The choice of pathogens was based on major fungal, bacterial, and oomycete pathogens of ornamental plants tested in our study. Based on our knowledge of pathogens and their damage and the promising antimicrobial properties of fire ant venom alkaloids, the overarching goal of this study was to develop an alternative management practice for pathogens in the ornamental plant production system. The objectives were to identify how effectively fire ant venom alkaloids and an alarm pheromone analog work against major pathogens of ornamental plants and what rate works best both in in vivo and in vitro assays.

## 2. Results

### 2.1. Test of Venom Alkaloids and Alarm Pheromone against Plant Fungal and Oomycete Pathogens

The venom alkaloid extract had a stronger inhibitory effect on pathogens than the alarm pheromone and control (acetone) (Table 1). All screened plant fungal and oomycete pathogens were inhibited by the venom alkaloids extract compared to the control (Table 1). The alarm pheromone showed significantly greater inhibition in *P. nicotianae* and *S. rolfsii* compared to the controls, and the effect was similar to that of the venom alkaloids. All application rates (2.4 µg/µL, 3.0 µg/µL, 3.6 µg/µL, and 4.8 µg/µL) of venom alkaloids were effective at inhibiting the growth of plant pathogens compared to the control (acetone 5 µL). *B. cinerea*, *F. oxysporum*, *P. citrinum*, and *R. solani* showed greater inhibition at 3 and 3.6 µg/µL venom alkaloids concentration compared to the 2.4 µg/µL concentration (Table 1). There was no proportionate increase in the zone of inhibition with increasing concentrations of venom alkaloids in the other tested pathogens.

### 2.2. Test of Venom Alkaloids and Alarm Pheromone against Plant Bacterial Pathogens

Venom alkaloids and alarm pheromone treatments numerically had larger zones of inhibition compared to the control when tested against *X. axonopodis* at 2.4 µg/µL concentration, but not with any other higher concentrations. For all the bacteria, neither the venom alkaloids nor alarm pheromone were effective at inhibiting colony growth and, therefore, statistics are not presented.

### 2.3. Test of Stability Effect of Venom Alkaloids against Plant Fungal and Oomycete Pathogens

**Figure 1:**
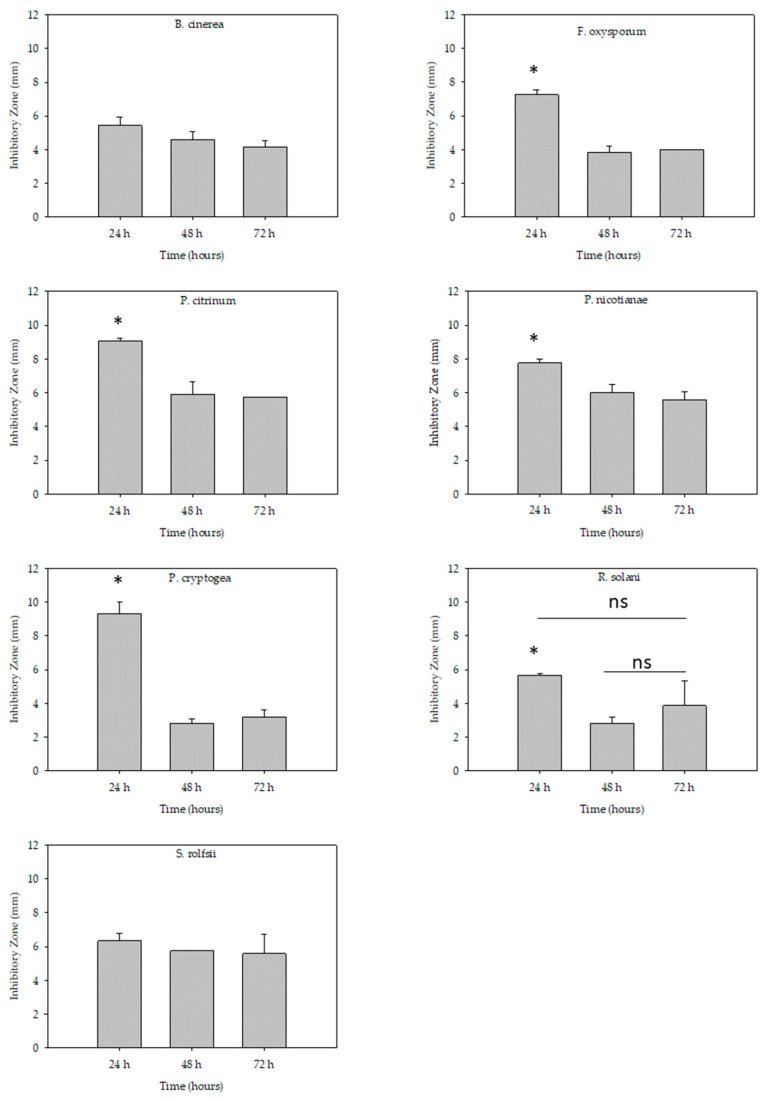
The stability effect of red imported fire ant venom alkaloids extracts to inhibit fungal and oomycete pathogens’ mycelium growth using the paper disc method. The inhibitory zones were evaluated 24 h, 48 h, and 72 h after 3 µg/mL treatment application. The bar represents the means ± SEM (mm). Means with symbol * are significantly different, according to Tukey’s Multiple Comparison Test at *p* < 0.05. The abbreviation ‘ns’ in the figure represents ‘not significant’ statistically at *p* < 0.05.

There were no differences in inhibitory zones between 24 h, 48 h, and 72 h periods in the case of *B. cinerea* and *S. rolfsii* (Figure 1). However, other fungal and oomycete pathogens had larger inhibitory zones at the 24 h evaluation time compared to 48 h and 72 h evaluation time (Figure 1). The exception was *R. solani* where the effect was not significant between 24 h and 72 h and between 48 h and 72 h but differ in the inhibition zone between 24 h and 48 h.

### 2.4. Greenhouse Bioassay against Soilborne Plant Pathogens

#### 2.4.1. *Rhizoctonia solani*

Damping-off percentage was significantly reduced when venom alkaloid concentrations above 0.15 µg/mL were used to drench inoculated pots (χ^2^ _(6,62)_ = 33.6, *p* < 0.001; Table 2). The inoculated control plants had higher root rot severity, which gradually decreased with increasing rates of venom alkaloids (χ^2^ _(6,62)_ = 321.69, *p* < 0.001). Both total plant and root weight were higher in venom alkaloid drenched plants (total plant weight: χ^2^ _(6,62)_ = 26.34, *p* < 0.001; root weight: χ^2^ _(6,62)_ = 35.09, *p* < 0.001). Pathogen recovery from sampled roots was lower in venom alkaloid drenched plants compared to the inoculated controls (χ^2^_(6,62)_ = 174.29, *p* < 0.001). This experiment was repeated twice in two trials and there was no effect of trials on damping-off (χ^2^ _(1,62)_ = 0.07, *p* = 0.79), root rot severity (χ^2^ _(1,62)_ = 0.19, *p* = 0.662), root weight (χ^2^ _(1,62)_ = 2.85, *p* = 0.091), and pathogen recovery (χ^2^ _(1,62)_ = 3.57, *p* = 0.068). However, total plant weight was significantly higher in the first trial compared to the second trial (χ^2^ _(1,62)_ = 7.44, *p* = 0.006).

#### 2.4.2. *Fusarium oxysporum*

Damping-off was reduced when venom alkaloids were drenched in inoculated pots (χ^2^_(6,62)_ = 27.66, *p* < 0.001; Table 3). The inoculated control plants had the highest root rot severity. Percentage of root rot gradually decreased with increasing rates of venom alkaloids (χ^2^ _(6,62)_ = 119.68, *p* < 0.001). Both plant weight and root weight were higher in venom alkaloid drenched plants (total plant weight: χ^2^ _(6,62)_ = 30.71, *p* < 0.001; root weight: χ^2^ _(6,62)_ = 33.15, *p* < 0.001) especially with 30 µg/mL and 3 µg/mL rates (Table 4). Pathogen recovery from sampled roots was lower in venom alkaloid drenched plants compared to the inoculated control plants (χ^2^ _(6,62)_ = 115.27, *p* < 0.001). This experiment was repeated twice in two trials and there was no effect of trials on damping-off (χ^2^ _(1,62)_ = 0.43, *p* = 0.512), root weight (χ^2^ _(1,62)_ = 0.30, *p* = 0.584), and pathogen recovery (χ^2^ _(1,62)_ = 0.03, *p* = 0.861). However, total plant weight was significantly higher in the first trial compared to the second trial (χ^2^ _(1,62)_ = 5.73, *p* = 0.017). Similarly, root rot severity was higher in the first trial compared to the second trial (χ^2^ _(1,62)_ = 12.62, *p* = 0.001).

#### 2.4.3. *Phytophthora nicotianae*

Damping-off was reduced when venom alkaloids were drenched in inoculated pots (χ^2^ _(6,62)_ = 55.99, *p* < 0.001; Table 4). The inoculated control plants had greater root rot severity, which decreased with increasing rates of venom alkaloids (χ^2^ _(6,62)_ = 331.02, *p* < 0.001). Some differences were observed in total plant weight (χ^2^ _(6,62)_ = 58.29, *p* < 0.001) and root weight (χ^2^_(6,62)_ = 57.97, *p* < 0.001) between the control and the venom alkaloid drenched plants. Total plant weight and root weight were higher in higher venom alkaloid rates compared 0.3 µg/mL and 0.15 µg/mL (Table 5). Pathogen recovery from sampled roots was lower in venom alkaloid drenched plants compared to the inoculated control plants (χ^2^ _(6,62)_ = 126.01, *p* < 0.001). This experiment was repeated twice in two trials and there was no effect of the trials on damping-off (χ^2^ _(1,62)_ = 0.34, *p* = 0.561), root rot severity (χ^2^ _(1,62)_ = 0.5, *p* = 0.479), root weight (χ^2^ _(1,62)_ = 3.59, *p* = 0.068), and pathogen recovery (χ^2^ _(1,62)_ = 0.13, *p* = 0.719). However, total plant weight was significantly higher in the first trial compared to the second trial (χ^2^ _(1,62)_ = 5.45, *p* = 0.019).

#### 2.4.4. *Botrytis cinerea*

Botrytis blight severity was reduced in venom alkaloid treated plants compared to the inoculated control (χ^2^ _(6,62)_ = 142.88, *p* < 0.001; Table 5). Phytotoxicity was higher in venom alkaloid sprayed plants compared to the control, with damage increasing with increasing concentration of venom alkaloids (χ^2^ _(6,62)_ = 396.61, *p* < 0.001). No differences in total plant weight were observed 30 µg/mL and 3 µg/mL and non-inoculated control (Table 5). However, the inoculated control had lower total plant weight compared to treatment and non-inoculated control (Table 5. Disease incidence in incubated leaf samples was lower in venom alkaloid sprayed plants compared to the inoculated control (χ^2^ _(6,62)_ = 331.72, *p* < 0.001) with decreasing disease incidence with increasing rates of venom alkaloids (Table 5. This experiment was repeated twice in two trials and there was no effect of trial on Botrytis blight severity (χ^2^ _(1,62)_ = 0.47, *p* = 0.494), disease incidence (χ^2^ _(1,62)_ = 0.48, *p* = 0.489), phytotoxicity (χ^2^ _(1,62)_ = 2.23, *p* = 0.135), and total plant weight (χ^2^ _(1,62)_ = 2.61, *p* = 0.075).

## 3. Discussion

Plant pathogens are a serious problem in food, fruits, and the production of ornamentals. There are many cultural, chemical, biological, biotechnical, and integrated management techniques available to control plant diseases. However, the economic, technical, environmental, and socio-cultural constraints remain a barrier to their success. For example, the use of resistant varieties or biological antagonists such as *Trichoderma* or *Pseudomonas* have been widely used all over the world. However, the host specificity of such control measures or difficulties in mass production have limited their usability. Therefore, additional products are required to prevent as well as control serious pathogens. One such key alternative is natural products, many of which have shown promising results in many aspects of crop management sciences. Our study found that red imported fire ant venom alkaloids are effective at reducing key soilborne and foliar pathogens. Therefore, this kind of natural product can be useful in formulating new chemicals or pesticides.

The alarm pheromone used in our study is a synthetic chemical analog of the fire ant alarm pheromone. There has been no prior research on the effect of this alarm pheromone on plant pathogens. However, it has been demonstrated that the mixture of alarm pheromone and venom alkaloids act synergistically to attract phorid flies, a specific parasitoid of fire ants, compared to either chemicals [36]. Therefore, the alarm pheromone was also included in both in vitro and in vivo assays. However, no noticeable effect of the alarm pheromone was observed in both assays. The chemical alarm pheromone contains 2-ethyl-3,6-dimethyl pyrazine [37] and was found to be ineffective at inhibiting the growth of the pathogens tested in our study.

In our study, we tested venom alkaloids against different pathogens in in vitro and in vivo assays. Venom alkaloids have shown positive results in controlling fungal and oomycete pathogens in both in vitro and in vivo assays. It has been previously tested for *Pythium* spp. and similar effects were observed [26]. However, they separated the venom alkaloids into two classes of compounds, piperidine, and piperideine, and thus tested each class separately against one oomycete pathogen. They did not find a difference in effect between the two classes of compounds. Therefore, the whole venom alkaloid extract was used in our study, but tested against oomycete, fungal, and bacterial pathogens. In addition, we do not know which compound in the extract is most effective against the plant pathogens tested here, however, the ratio of the major alkaloid components is reported in the methods (see Section 4.1). Of the five major alkaloids, dehydrosolenopsin B (C13:1) was the dominant compound, representing 72% of the alkaloid profile. A range of alkaloid rates were tested and we found that the lowest rate was still enough to prevent the fungal and oomycete pathogens growth. In some pathogens (e.g., *B. cinerea*, *F. oxysporum*, *P. citrinum*, and *R. solani*), venom alkaloid rates 3 and 3.6 µg/µL showed higher inhibition compared to the 2.4 µg/µL. However, other tested soilborne pathogens had somewhat similar inhibition regardless of the venom alkaloid rate. This shows that the lower amount of venom alkaloid is enough to prevent the growth of pathogens. The stability test adds to the promising characteristic of venom alkaloids. In most cases, the effect was either constant until 3 days (72 h) of the testing period or strong enough to prevent pathogen growth. The reason why the inhibitory zone may be larger at 24 h compared to 48 h and 72 h could be due to the lack of optimal growth of pathogens in the early time interval. Many fungal pathogens take more than 24 h to fully grow. Therefore, venom alkaloids were significant enough to inhibit pathogens over a longer test period when pathogen growth should have been optimal. The test on *Pythium* spp. confirmed the stability of the piperidine alkaloid inhibition from 2 to 12 weeks in different sets of storage temperature [26].

Greenhouse assays were also carried out with impatiens plants to evaluate the inhibitory effect of venom alkaloids on fungal and oomycete pathogens. Four different assays were carried out, three with soilborne pathogens and one with a foliar pathogen. The venom alkaloid extract treatment improved crop health parameters in the presence of soilborne pathogens. For example, root rot severity, damping-off, and pathogen recovery were higher in inoculated control compared to the venom-treated plants. There was some percentage of disease recovery in water and acetone only controls, which was not expected. The possible explanation for that would be splash dispersal or contamination during irrigation or by other means. In a few cases, the efficacy of the venom alkaloid treatment decreased with decreasing rates of venom alkaloids. The rate used in the greenhouse assay was diluted 10, 100, and 200 times compared to the in vitro assay and it was expected that higher dilutions might be less effective. The study conducted by Li et al. [26] with piperideine alkaloid against *Pythium* spp. found that venom alkaloids were able to reduce pathogen mycelial growth and sporulation. They also found a comparable increase in cucumber plant emergence and plant height with drenched venom alkaloid compared to controls. Our greenhouse assays were conducted in two trials and, in all tested fungal pathogens, a higher total plant weight was observed in the first trial compared to the second trial. The root weight and root rot severity were similar across the trials, which eliminates the possible effect of root health on total plant weight in our study. The possible explanation for that is the time of trials conducted. The first and second trials were conducted in a month interval (first trial on June 29–July 21 and a second trial on July 28–August 21) and even though they were both carried out in a greenhouse, the seasonal effect might have caused differences in total plant weight. Further research is needed to evaluate this effect on total plant weight.

Venom alkaloids not only suppressed soilborne pathogens but also decreased the severity of Botrytis blight caused by *B. cinerea*. However, the phytotoxicity effect of higher rates of venom alkaloids could not be avoided. The higher dilution rates of venom alkaloids were also less effective at protecting against foliar blight disease. However, lower phytotoxicity occurred at diluted rates compared to the higher concentrate rates. Therefore, it is crucial to find the optimum rate of venom alkaloids or improve formulation to minimize phytotoxicity and aesthetic injury. The market of ornamental nurseries always tries to avoid aesthetic injury because injured plants are difficult to sell. The disease incidence conducted after one week of incubation showed promising results of controlling foliar disease for an extended period. Overall, Botrytis blight disease incidence level was lower on plants treated with venom alkaloids.

There was no effect of venom alkaloid against bacterial pathogens. The test carried out by Sullivan et al. [38] using nine different synthetic venom alkaloids showed a few promising results in controlling *Streptococcus pneumoniae* but failed to control *Enterococcus faecalis*, *Escherichia coli*, and *Pseudomonas aeruginosa*. All the bacteria tested by Sullivan et al. were human pathogens. Another study carried out by Jouvenaz et al. [34] found that Gram-positive bacteria were more susceptible to synthetic venom alkaloids compared to Gram-negative bacteria. In both previous studies, they used synthetic venom alkaloids and found variable results where some were bioactive but not others. The later study evaluated the effectiveness of these alkaloids just after 24 h of treatment and measured the inhibitory zone. Sometimes bacteria take a longer period to grow, so a longer bioassay interval may be more informative. We evaluated the inhibitory zone after 48 h. The test carried out in our study was against plant bacterial pathogens and no reduction in growth was observed over the two-day period.

The inhibitory effect of venom alkaloid used in both in vitro and in vivo assays were clear enough to see compared to the controls. The increasing losses of plant stock from soilborne and foliar pathogens in ornamental plants can be managed by the application of venom alkaloids. However, it is crucial to find optimum rates of venom alkaloids for both soilborne and foliar pathogens and to develop formulations that minimize phytotoxicity. In conclusion, venom alkaloids can be a source of new control measures or can be incorporated in fungicides and/or respective pathogens’ controlling formulations. The formulations of such products and range of pathogen efficacy will require further testing.

## 4. Materials and Methods

### 4.1. Fire Ant Collection, Venom Alkaloids Extraction

Hybrid imported fire ants, *Solenopsis invicta × richteri,* were collected from colonies across middle Tennessee, USA. Approximately 100 ants from each colony were transferred to 20-mL scintillation vials and ants were submerged in hexane for 24 h to collect venom secretions. Extracts from 500 colonies were combined and concentrated under nitrogen. The venom extract was analyzed using GC-MS (Shimadzu QP-2010, Shimadzu, Kyoto, Japan) to determine the proportions of major venom alkaloids in the extract (Solenopsin A (C:11) = 0.04, Dehydrosolenopsin B (C13:1) = 0.72, Solenopsin B (C13) = 0.10, Dehydrosolenopsin C (C15:1) = 0.13, Solenopsin C (C15) = 0.01; [29]. Other minor alkaloid components were also present in the extract. The extract was stored at −20 °C until use in the bioassays.

### 4.2. Fungal, Oomycetes, and Bacterial Culture Preparation

Fungal and oomycete pathogen isolates (*B. cinerea, F. oxysporum*, *P. cryptogea*, *P. citrinum*, *P. nicotianae*, *R. solani*, and *S. rolfsii*) and bacterial pathogen isolates (*P. syringae, X. axonopodis*, and *X. campestris*) were used in these experiments. All isolates were acquired from Dr. Fulya Baysal-Gurel’s culture collection at the Tennessee State University Nursery Research Center (TSUNRC) in McMinnville, TN. Fungal and oomycete pathogens were isolated from diseased impatiens plants in 2016 and maintained on potato dextrose agar (PDA) medium (Sigma-Aldrich, St. Louis, MO, USA), whereas isolates of bacterial pathogens were maintained on nutrient broth yeast agar (NBYA) medium. The confirmation of virulence was carried out before the greenhouse experiments by inoculating impatiens plants with all pathogens and re-isolating them. To prepare cultures for inoculation, each culture was incubated at 25 °C for 7–10 days for fungal and oomycete pathogens and 2–3 days for bacterial pathogens.

### 4.3. Test of Venom Alkaloids and Alarm Pheromone against Plant Fungal and Oomycete Pathogens

Each pathogen was tested for the inhibitory effect of both the venom alkaloids extract and alarm pheromone. The test of inhibitory activity was carried out by the method used in Li et al. [26]. Briefly, 5 mm discs of Fisherbrand qualitative-grade filter paper (Thermo Fisher Scientific, Waltham, MA, USA) were cut and sterilized at 121 °C for 30 min. Four filter paper discs were used for each 9 cm PDA plate. Before placing it on a PDA plate, 5 µL of an acetone solution containing either the venom alkaloids extract or alarm pheromone was applied to the filter paper. The papers were held under a laminar flow hood until the acetone solvent evaporated. Four rates of venom alkaloids extract or alarm pheromone were prepared and tested against each pathogen. Rates of both treatments were 2.4 µg/µL, 3 µg/µL, 3.6 µg/µL, and 4.8 µg/µL. For example, at the rate of 2.4 µg/µL, a total of 12 µg of alkaloid or alarm pheromone were applied in the 5 µL of acetone. Each PDA treatment plate was arranged with a 5 mm plug of the pathogen at the center and four treated paper discs 2 cm away from the pathogen at 0, 90, 180, 270° positions, respectively. The control plates had paper discs with 5 µL of acetone solvent applied. Each treatment and control were replicated three times. Plates were incubated at 25 °C for 48 h in darkness. Inhibition zones (the linear distance from the growing edge of the colony to the treated paper disc) were measured in all four directions. The rates of venom alkaloid were chosen based on the finding by Li et al. [26] and were found effective against tested pathogen. They used 5 µL of an acetone solution of piperidine (3.8 µg/µL) or piperideine (3.8 µg/µL) alkaloids in their study against *P. ultimum*. In addition, our study used 4 different rates in an assumption that even a lower rate than 3.8 µg/µL could be effective to control other fungal and oomycete pathogen.

### 4.4. Test of Stability Effect of Venom Alkaloids against Fungal Pathogens and Oomycete Pathogens

The 3 µg/µL rate was used to test the stability effect of the venom alkaloids extract. Inhibition zones were measured at 24 h, 48 h, and 72 h after treatment application. Plates were incubated at 25 °C in darkness and measured for inhibition zones after 24, 48, and 72 h. Every treatment had three replications.

### 4.5. Test of Venom Alkaloids and Alarm Pheromone against Bacterial Pathogens

To prepare bacterial treatment bioassay plates, a bacterial suspension at 0.2 optical density (OD) was prepared. A 100 µL of suspension was disbursed on NYBA medium using 3 mm solid glass beads (Walter Stern Inc., Port Washington, NY, USA) so that the solution was distributed evenly over the plate. Following the same procedure for the fungal and oomycete assays, four 5 mm paper discs treated with either venom alkaloids extract or alarm pheromone were placed at 2 cm away from the center of the plate at 0, 90, 180, 270°, respectively. The control plate received filter paper discs with 5 µL solution of acetone solvent applied. Plates were incubated for 48 h at 25 °C and inhibition zones were measured. Because bacterial pathogens were evenly distributed in the NYBA medium before placing the paper discs, the inhibitory zone was calculated by measuring the linear distance between paper discs and growing edge in all four directions for each paper disc. Therefore, there were 16 measurements for each plate. Each treatment and control were replicated three times.

### 4.6. Greenhouse Bioassays

The greenhouse experiment was conducted at the TSUNRC in McMinnville, TN. Bacterial pathogens were excluded from the greenhouse assay since no significant inhibitory effect was observed when treated with both alarm pheromone and venom alkaloids in the in vivo assays. Although the venom alkaloids had an inhibitory effect on all fungal and oomycete pathogens tested, only *B. cinerea*, *F. oxysporum*, *P. nicotianae*, and *R. solani* were selected to perform greenhouse bioassays on impatiens (*Impatiens walleriana* ‘Super Elfin XP Violet’). Seeds were obtained from Harris Seeds/Garden Trends, Inc. (Rochester, NY, USA). Impatiens plant can serve as a host to the *R. solani* [39], *B. cinerea* [40], *P. nicotianae* [41,42], and *F. oxysporum* [43]. With the exception of *B. cinerea* (foliar pathogen), all other pathogens are soilborne pathogens, and therefore, two sets of greenhouse bioassays were conducted. All greenhouse experiments included a positive (+pathogen), negative (non-inoculated) control, and acetone solvent treatment. Greenhouse assays were conducted twice. Seed sowing was carried out on 29 June 2017 and 28 July 2017 and plant health parameters were evaluated on 21 July 2017 and 21 August 2017 respectively for the first and second trials.

#### 4.6.1. Soilborne Pathogens

Impatiens seeds were tested for germination before greenhouse bioassay. Germination was 100% in water agar plates at 22 °C. Potting mix (Morton’s Grow Mix #2: Canadian sphagnum peat [60%], vermiculite [20%], and perlite [20%], with an average substrate bulk density reported by the manufacturer of 144 kg/m^3^) (Morton’s Horticultural Products, McMinnville, TN, USA) was used to grow impatiens seeds in the greenhouse. The potting mix was sterilized using an electric soil sterilizer (Pro-Grow Supply Corp., Model SS-30 Brookfield, WI, USA) before use. All three soilborne pathogens were cultured on PDA media as previously described and stored in darkness at room temperature for one week. Nursery containers (7 × 5 × 5 cm^3^) were used and the sterile potting mix was filled up to 2 cm below the top of the pot. Five seeds (one at the center and four at the 0, 90, 180, and 270°, respectively) were placed on the potting mix (29 June 2017–28 July 2017) and two 5 cm excised pathogen discs from the colony growing edge were transferred to the potting mix, then the surface was covered with 1 cm of sterile potting mix.

Four rates of venom alkaloids were used in the greenhouse bioassay (30 µg/mL, 3 µg/mL, 0.3 µg/mL, and 0.15 µg/mL). The venom alkaloid extract was first diluted with 1 mL of acetone and then mixed with water to make 10 mL of total volume. Two drops of Tween-80 (Sigma-Aldrich, St. Louis, MO, USA) were mixed with 10 mL of solution and applied as a drench to the surface of the potting mix. Control pots received 1 mL of acetone, 9 mL of water, and 2 drops of tween-80. Treated pots were placed in a tray (each tray can hold 15 pots) and transferred to greenhouse benches. The greenhouse temperature was maintained at 27 and 21 ± 2 °C, day and night with 85% relative humidity and 14 h day length for one month. Each treatment and control were replicated five times.

#### 4.6.2. Foliar Pathogen

Similar methods were followed as described for soilborne pathogens except for the treatment application method. Venom alkaloids and control treatments were applied to foliage until runoff using a backpack CO_2_-pressurized sprayer (Bellspray, Opelousas, LA, USA) with a tapered edge flat spray pattern stainless steel nozzle (TP8002VS; TeeJet Technologies, Springfield, IL, USA) at 40 psi. Treatments were applied inside the greenhouse 15 days after inoculation with *B. cinerea*. Each treatment and control were replicated five times.

### 4.7. Evaluation of Crop Health

#### 4.7.1. Soilborne Pathogens

Germination count was recorded 1 week after seeding. The root weight and plant weight were measured for each potted plant at the completion of the greenhouse bioassay. Total plants dead due to post-emergence damping-off and plants not germinated were recorded to calculate the percentage of damping-off for soilborne pathogens. All plants after one month of seeding were removed from the pot and rinsed for 30 min under tap water to evaluate the root health. A visual assessment was carried out to calculate the root rot severity using a scale of 0%–100% of the total root system affected by soilborne pathogens. Ten randomly selected root samples (1 cm long) from each treatment were plated on selective media for each soilborne pathogen and incubated at 25 °C in darkness. After 3 days, the total number of roots showing pathogen growth were counted and the pathogen recovery percentage was calculated by dividing infected roots by total root pieces plated times 100.

#### 4.7.2. Foliar Pathogen

The effect of phytotoxicity due to treatment spray was evaluated on a scale of 0%–100% of the total leaf area affected one week after treatment. Plants were rated for plant disease severity by using a scale of 0%–100% of the total plant (above crown) system affected by the foliar pathogen. Total plant weight was measured. At the end of each bioassay, three leaves from each treatment were sampled randomly and kept in a plastic incubation chamber with a wet paper towel at 25 °C in darkness. After one week, leaves were assessed for disease incidence and rated from 0%–100% of the individual leaf affected.

### 4.8. Statistical Analysis

The means of the inhibitory zone for each treatment, pathogen, and rates were calculated using PROC MEANS (SAS 9.4, SAS Institute, Inc., Cary, NC, USA). A generalized linear model (PROC GENMOD) sorted by pathogen and fitted to a normal distribution was used to calculate whether the inhibitory zone was different between treatments and controls. The stability test of venom alkaloid was analyzed similarly, using PROC GENMOD sorted by pathogens to see the difference between 24 h, 48 h, and 72 h after treatment (Inhibition = Time Trial). The least Square (LS) means were separated by Tukey’s Multiple Comparison Test at α = 0.05. Finally, the means for greenhouse bioassay were calculated using PROC MEANS sorted by pathogens and rates applied. The crop health factors (damping-off, root rot severity, total plant weight, root weight, pathogen recovery, phytotoxicity, disease incidence, Botrytis foliar blight severity) were analyzed in the same manner as inhibition, sorted by pathogens where LS means were separated by Tukey’s Multiple Comparison Test at α = 0.05.

## Figures and Tables

**Table 1:** Inhibitory zone (mm) ± SEM measured for different fungal and oomycete pathogen plates when treatments (venom alkaloid and alarm pheromone) were tested at different rates and compared to the control (acetone).

Pathogen	Treatment	Inhibitory Zone (mm) ± SEM				Within Treatment Statistics
		2.4 µg/µL *	3.0 µg/µL	3.6 µg/µL	4.8 µg/µL	
*B. cinerea*	Venom	0.7 ± 0.3 ^a^ ** (C)	2.4 ± 0.2 ^a^ (A)	1.4 ± 0.7 ^a^ (B)	2.3 ± 0.4 ^a^ (A)	χ^2^_(3,8)_ = 10.61, *p* = 0.014
	Alarm	0 ^b^	0 ^b^	0 ^b^	0 ^b^	
	Control ***	0 ^b^				
	Between treatment statistics	χ^2^_(2,6)_ = 9.85, *p* = 0.007	χ^2^ _(2,6)_ = 240.29, *p* < 0.001	χ^2^_(2,6)_ = 7.92, *p* = 0.019	χ^2^ _(2,6)_ = 82.53, *p* < 0.001	
*F. oxysporum*	Venom	0.6 ± 0.2 ^a^ (B)	1.9 ± 0.2 ^a^ (A)	2.0 ± 0.3 ^a^ (A)	1.9 ± 0.2 ^a^ (A)	χ^2^_(3,8)_ = 26.77, *p* < 0.001
	Alarm	0 ^b^	0 ^b^	0 ^b^	0 ^b^	
	Control	0 ^b^				
	Between treatment statistics	χ^2^ _(2,6)_ = 14, *p* = 0.001	χ^2^ _(2,6)_ = 151.14, *p* < 0.001	χ^2^ _(2,6)_ = 96, *p* < 0.001	χ^2^ _(2,6)_ = 264.5, *p* < 0.001	
*P. citrinum*	Venom	3.8 ± 0.7 ^a^ (B)	5.0 ± 0.0 ^a^ (A)	5.0 ± 0.0 ^a^ (A)	4.7 ± 0.3 ^a^ (AB)	χ^2^_(3,8)_ = 6.85, *p* = 0.077
	Alarm	0 ^b^	0 ^b^	0 ^b^	0 ^b^	
	Control	0 ^b^				
	Between treatment statistics	χ^2^ _(2,6)_ = 54, *p* < 0.001	χ^2^ _(2,6)_ = 50, *p* < 0.001	χ^2^ _(2,6)_ = 50, *p* < 0.001	χ^2^ _(2,6)_ = 482.46, *p* < 0.001	
*P. cryptogea*	Venom	0.6 ± 0.3 ^a^ (B)	0.8 ± 0.1 ^a^ (AB)	1.2 ± 0.1 ^a^ (A)	1.1 ± 0.2 ^a^ (AB)	χ^2^_(3,8)_ = 6.32, *p* = 0.097
	Alarm	0 ^b^	0 ^b^	0 ^b^	0 ^b^	
	Control	0 ^b^				
	Between treatment statistics	χ^2^ _(2,6)_ = 7.54, *p* = 0.023	χ^2^ _(2,6)_ = 200, *p* < 0.001	χ^2^ _(2,6)_ = 392, *p* < 0.001	χ^2^ _(2,6)_ = 84.50, *p* < 0.001	
*P. nicotianae*	Venom	2.8 ± 0.5 ^a^	2.7 ± 0.2 ^a^	2.3 ± 0.5 ^a^	3.2 ± 0.2 ^a^	χ^2^_(3,8)_ = 2.26, *p* = 0.521
	Alarm	0.4 ± 0.4 ^b^	0.3 ± 0.1 ^b^	0.5 ± 0.1 ^b^	0.5 ± 0.1 ^b^	
	Control	0 ^b^				
	Between treatment statistics	χ^2^ _(2,6)_ = 29.66, *p* < 0.001	χ^2^ _(2,6)_ = 288, *p* < 0.001	χ^2^ _(2,6)_ = 32.6, *p* < 0.001	χ^2^ _(2,6)_ = 250.4, *p* < 0.001	
*R. solani*	Venom	1.3 ± 0.3 ^a^ (B)	2.4 ± 0.3 ^a^ (A)	2.5 ± 0.2 ^a^ (A)	2.5 ± 0.1 ^a^ (A)	χ^2^_(3,8)_ = 12.46, *p* = 0.006
	Alarm	0 ^b^	0 ^b^	0 ^b^	0 ^b^	
	Control	0 ^b^				
	Between treatment statistics	χ^2^ _(2,6)_ = 37.50, *p* < 0.001	χ^2^ _(2,6)_ = 105.12, *p* < 0.001	χ^2^ _(2,6)_ = 600, *p* < 0.001	χ^2^ _(2,6)_ = 85.71, *p* < 0.001	
*S. rolfsii*	Venom	3.8 ± 0.7 ^a^ (B)	5.3 ± 0.3 ^a^ (A)	3.5 ± 0.4 ^a^ (B)	4.7 ± 0.5 ^a^ (AB)	χ^2^_(3,8)_ = 8.64, *p* = 0.035
	Alarm	1.3 ± 0.5 ^b^	0.7 ± 0.2 ^b^	0.9 ± 0.4 ^b^	0.7 ± 0.1 ^b^	
	Control	0 ^b^				
	Between treatment statistics	χ^2^ _(2,6)_ = 31.82, *p* < 0.001	χ^2^ _(2,6)_ = 317.22, *p* < 0.001	χ^2^ _(2,6)_ = 61.87, *p* < 0.001	χ^2^ _(2,6)_ = 144.84, *p* < 0.001	

* The tested rates of venom alkaloid and alarm pheromone were 2.4 µg/µL, 3.0 µg/µL, 3.6 µg/µL, and 4.8 µg/µL and compared to the control (acetone 5 µL). For example, at the rate of 2.4 µg/µL, 12 µg of either alkaloid or alarm pheromone was present in 5 µL of acetone. ** Means that do not share a letter are significantly different, according to Tukey’s Multiple Comparison Test at *p* < 0.05. Capital letters in parentheses represent within the treatment significance whereas small letters represent between treatments significance. *** Control did not have any rates. Treatments were compared to the control receiving 5 µL acetone.

**Table 2:** Impatiens crop health parameters (damping-off, Rhizoctonia root rot severity, total plant weight, root weight, and pathogen recovery) (± SEM) were assessed in a greenhouse experiment and compared to *Rhizoctonia solani* inoculated control. This experiment was repeated twice.

Treatment	Descriptions (Rates of Alkaloid *)	Damping-Off (%)	Root Rot Severity (%)	Total Plant Weight (g)		Root Weight (g)	Pathogen Recovery (%)
				1st Trial ***	2nd Trial		
Venom	30 µg/mL + pathogen	6 ± 3.1 ^b^ **	5 ± 3.8 ^c^	14.1 ± 1.3 ^a^	11.7 ± 1.8 ^a^	3.1 ± 0.5 ^ab^	19 ± 3.5 ^c^
	3 µg/mL + pathogen	4 ± 2.7 ^b^	1 ± 0.6 ^cd^	12.0 ± 1.2 ^ab^	11.0 ± 1.8 ^a^	2.6 ± 0.4 ^a^	19 ± 2.8 ^c^
	0.3 µg/mL + pathogen	4 ± 2.7 ^b^	26 ± 5.1 ^b^	10.3 ± 1.6 ^ab^	7.2 ± 0.7 ^b^	1.8 ± 0.3 ^cd^	34 ± 8.1 ^c^
	0.15 µg/mL + pathogen	12 ± 6.7 ^b^	31 ± 5.0 ^b^	10.1 ± 1.5 ^ab^	8.0 ± 0.7 ^b^	2.2 ± 0.4 ^bc^	54 ± 9.8 ^b^
Control	+ pathogen	40 ± 7.4 ^a^	64 ± 2.2 ^a^	7.7 ± 1.3 ^b^	5.0 ± 0.8 ^b^	0.8 ± 0.1 ^d^	99 ± 1.0 ^a^
	Water only	10 ± 10.0 ^b^	2 ± 1.3 ^cd^	12.1 ± 2.5 ^ab^	11.1 ± 2.9 ^a^	3.7 ± 0.6 ^a^	17 ± 6.5 ^c^
	Acetone only	0 ^b^	0 ^cd^	13.5 ± 1.9 ^a^	10.0 ± 1.1 ^a^	3.1 ± 0.5 ^a b^	11 ± 4.6 ^c^

* The tested rates of red imported fire ant venom alkaloids were 30 µg/mL, 3.0 µg/mL, 0.3 µg/mL, and 0.15 µg/mL and compared to the control. ** Means that do not share a letter are significantly different, according to Tukey’s Multiple Comparison Test at *p* < 0.05. *** Experiment was conducted in two trials (twice). If the trial value is significant, results are presented separately for each trial. However, if not, pooled mean of two trials are presented in the table.

**Table 3:** Impatiens crop health parameters (damping-off, Fusarium root rot severity, total plant weight, root weight, and pathogen recovery) (± SEM) were assessed in a greenhouse experiment and compared to *Fusarium oxysporum* inoculated control. This experiment was repeated twice.

Treatment	Descriptions (Rates of Alkaloid *)	Damping-Off (%)	Root Rot Severity (%)		Total Plant Weight (g)		Root Weight (g)	Pathogen Recovery (%)
			1st Trial ***	2nd Trial	1st Trial	2nd Trial		
Venom	30 µg/mL + pathogen	14 ± 4.3 ^ab^ **	0 ^c^	3 ± 1.5 ^bc^	12.0 ± 1.3 ^a^	12.7 ± 0.8 ^a^	3.1 ± 0.4 ^a^	18 ± 4.9 ^c^
	3 µg/mL + pathogen	10 ± 4.5 ^b^	8 ± 5.0 ^c^	0 ^c^	12.4 ± 1.8 ^a^	13.6 ± 1.4 ^a^	2.9 ± 0.5 ^a^	21 ± 4.1 ^c^
	0.3 µg/mL + pathogen	10 ± 3.3 ^b^	40 ± 2.5 ^b^	5 ± 2.3 ^b^	7.3 ± 0.8 ^b^	11.7 ± 0.3 ^ab^	1.8 ± 0.3 ^b^	40 ± 8.4 ^b^
	0.15 µg/mL + pathogen	4 ± 2.7 ^bc^	43 ± 8.5 ^b^	14 ± 5.0 ^b^	7.9 ± 1.1 ^b^	11.5 ± 1.7 ^b^	2.0 ± 0.4 ^b^	52 ± 10.8 ^b^
Control	+ pathogen	20 ± 5.2 ^a^	65 ± 13.9 ^a^	50 ± 7.9 ^a^	7.8 ± 0.9 ^b^	8.4 ± 0.9 ^b^	1.1 ± 0.1 ^b^	92 ± 2.9 ^a^
	Water only	0 ^c^	3 ± 2.5 ^c^	6 ± 4.8 ^b^	12.3 ± 3.6 ^a^	12.8 ± 2.2 ^a^	3.3 ± 0.4 ^a^	16 ± 5.8 ^c^
	Acetone only	0 ^c^	7.5 ± 7.5 ^c^	2.5 ± 2.5 ^b c^	12.8 ± 0.5 ^a^	13.0 ± 1.5 ^a^	2.9 ± 0.3 ^a^	14 ± 4.3 ^c^

* The tested rates of red imported fire ant venom alkaloids were 30 µg/mL, 3.0 µg/mL, 0.3 µg/mL, and 0.15 µg/mL and compared to the control. ** Means that do not share a letter are significantly different, according to Tukey’s Multiple Comparison Test at *p* < 0.05. *** Experiment was conducted in two trials (twice). If the trial value is significant, results are presented separately for each trial. However, if not, the pool mean of two trials is presented in the table.

**Table 4:** Impatiens crop health parameters (damping-off, Phytophthora root rot severity, total plant weight, root weight, and pathogen recovery) (±SEM) were assessed in a greenhouse experiment and compared to the *Phytophthora nicotianae* inoculated control. This experiment was repeated twice.

Treatment	Descriptions (Rates of Alkaloid *)	Damping-Off (%)	Root Rot Severity (%)	Total Plant Weight (g)		Root Weight (g)	Pathogen Recovery (%)
				1st Trial ***	2nd Trial		
Venom	30 µg/mL + pathogen	10 ± 4.5 ^cd^ **	0 ^d^	12.3 ± 0.9 ^ab^	12.2 ± 0.8 ^a^	3.3 ± 0.3 ^a^	18 ± 3.9 ^c^
	3 µg/mL + pathogen	6 ± 3.1 ^c^	3 ± 1.7 ^d^	11.7 ± 1.3 ^ab^	11.2 ± 1.3 ^a^	3.2 ± 0.4 ^a^	17 ± 5.0 ^c^
	0.3 µg/mL + pathogen	14 ± 5.2 ^bc^	25 ± 3.2 ^b^	10.6 ± 1.2 ^abc^	9.5 ± 0.5 ^bc^	2.0 ± 0.3 ^b^	27 ± 7.8 ^bc^
	0.15 µg/mL + pathogen	18 ± 3.6 ^b^	15 ± 6.1 ^c^	9.1 ± 1.2 ^bc^	6.6 ± 0.6 ^cc^	1.6 ± 0.2 ^b^	36 ± 9.6 ^b^
Control	+ pathogen	34 ± 6.0 ^a^	61 ± 2.9 ^a^	8.2 ± 1.3 ^c^	6.5 ± 0.6 ^c^	1.6 ± 0.2 ^b^	96 ± 3.1 ^a^
	Water only	0 ^d^	2 ± 1.3 ^d^	13.2 ± 1.1 ^a^	11.8 ± 0.6 ^a^	3.8 ± 0.3 ^a^	17 ± 6.2 ^c^
	Acetone only	0 ^d^	1 ± 1.3 ^d^	12.4 ± 1.4 ^a^	11.3 ± 0.5 ^a^	3.3 ± 0.4 ^a^	17 ± 6.0 ^c^

* The tested rates of red imported fire ant venom alkaloids were 30 µg/mL, 3.0 µg/mL, 0.3 µg/mL, and 0.15 µg/mL and compared to the control. ** Means that do not share a letter are significantly different, according to Tukey’s Multiple Comparison Test at *p* < 0.05. *** Experiment was conducted in two trials (twice). If the trial value is significant, results are presented separately for each trial. However, if not, pooled mean of two trials are presented in the table.

**Table 5:** Impatiens crop health parameters (Botrytis blight severity, phytotoxicity, total plant weight, and disease incidence) (± SEM) were assessed in a greenhouse experiment and compared to *Botrytis cinerea* inoculated control. This experiment was repeated twice.

Treatment	Descriptions (Rates of Alkaloid *)	Botrytis Blight Severity (%)	Disease Incidence (%)	Phytotoxicity (%)	Total Plant Weight (g)
Venom	30 µg/mL + pathogen	4 ± 1.9 ^c^ **	4 ± 1.1 ^c^	67 ± 3.2 ^a^	14.5 ± 1.8 ^a^
	3 µg/mL + pathogen	8 ± 2.6 ^bc^	4 ± 0.8 ^c^	25 ± 7.3 ^b^	15.2 ± 1.4 ^ab^
	0.3 µg/mL + pathogen	18 ± 7.2 ^b^	10 ± 2.3 ^b^	10 ± 1.6 ^c^	12.3 ± 1.6 ^b^
	0.15 µg/mL + pathogen	19 ± 6.0 ^b^	13 ± 2.5 ^b^	1 ± 0.7 ^d^	13.5 ± 2.5 ^b^
Control	+ pathogen	63 ± 8.3 ^a^	52 ± 5.2 ^a^	0 ^d^	9.2 ± 0.8 ^c^
	Water only	0 ^c^	1 ± 0.6 ^c^	0 ^d^	18.1 ± 1.6 ^a^
	Acetone only	0 ^c^	1 ± 0.4 ^c^	0 ^d^	14.9 ± 1.4 ^a^

* The tested rates of red imported fire ant venom alkaloids were 30 µg/mL, 3.0 µg/mL, 0.3 µg/mL, and 0.15 µg/mL and compared to the control. ** Means that do not share a letter are significantly different, according to Tukey’s Multiple Comparison Test at *p* < 0.05.

## Data Availability

Not applicable.
